# Potential guidelines for cataract surgery and rehabilitation in visually impaired patients: Literature analysis

**DOI:** 10.1002/agm2.12386

**Published:** 2024-12-13

**Authors:** Paolo Giuseppe Limoli, Celeste Limoli, Marcella Nebbioso

**Affiliations:** ^1^ Low Vision Research Centre of Milan Milan Italy; ^2^ Department of Sense Organs, Faculty of Medicine and Odontology, Rare Retinal Diseases and Ocular Electrophysiology Centre, Umberto I Policlinic Sapienza University of Rome Rome Italy

**Keywords:** intraocularf lens, low vision, near vision, neuroretinal diseases, phacoemulsification, visual rehabilitation

## Abstract

Cataracts can reduce the quality of vision in visually impaired patients who already have a visual impairment. The most common causes of low vision include age‐related macular degeneration (AMD), high myopia (HM), diabetic retinopathy (DR), glaucoma (GL), and inherited degenerative ocular diseases. The surgery aims to improve their independence, quality of life, and ability to engage in daily, social, and work activities. Phacoemulsification and intraocular lens (IOL) implantation, combined with visual rehabilitation, can improve visual acuity of visually impaired patients. Therefore, comprehensive guidelines for cataract surgery in patients with low vision would be beneficial to ensure optimal surgical outcomes by improving surgical planning, execution, and postoperative care, along with a well‐coordinated rehabilitation process. In cases of reduced metabolism, such as low vision, oxidative stress can be aggravated by light exposure and surgical interventions. Thus, maintaining redox balance is crucial for stabilizing retinal conditions. Patients with visual impairments rely on retinal regions with the greatest residual function, and cataract surgery aims to enhance focus on these areas, improving reading quality and reducing scotoma perception. Thorough informed consent is crucial, ensuring that patients are fully aware of the potential risks, benefits, and limitations of surgery. Close postoperative follow‐up in the first 6 months is crucial to detect and manage any complications promptly, such as reactivation of maculopathy. The aim of this work is to establish potential guidelines for optimal rehabilitation outcomes through careful literature analysis.

## Graphical abstract

Cataract phacoemulsification combined with the implantation of the most suitable intraocular lens can improve the visual acuity of visually impaired patients. Visual rehabilitation involves special tools such as microperimetry, prisms, lenses, electronic equipment, and specific neuromodulation techniques that stimulate the brain and neuroretina. This work exposes potential information for optimal rehabilitation outcomes through a careful review of the literature.

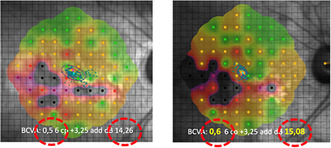

## 1 INTRODUCTION

Visual rehabilitation is a therapeutic process that aims to improve or visual function in individuals with compromised visual performance due to injury or disease. The primary objective is to enhance patients' independence, quality of life, and capacity to perform daily activities, as well as social and occupational tasks, with greater efficiency.^1^, ^2^

Visual rehabilitation focuses on three key aspects:^3^, ^4^

The recovery of residual visual function, adaptation to new visual conditions, and optimization of remaining vision through the enhancement of other sensory modalities. The most common etiologies of low vision include age‐related macular degeneration (AMD), high myopia (HM), diabetic retinopathy (DR), glaucoma (GL), and inherited degenerative ocular disorders.^5^ Effective visual rehabilitation often employs specialized devices, including prisms, lenses, and electronic aids, to assist individuals in maximizing their visual capabilities.^6^, ^7^

The rehabilitation process includes specialized neuromodulation techniques designed to stimulate the brain and neuroretina, facilitating the formation of new synapses, reducing postsynaptic inhibition, synchronizing neural signals, and minimizing “background noise.”^4^ Additionally, it employs pharmacological and cellular approaches aimed at promoting neuroenhancement in target cells. This is achieved through mitochondrial reactivation, attenuation of the inflammatory effects associated with retinal pathology, improvement of hemorheological properties, and stabilization of redox imbalances, ultimately mitigating cellular apoptosis.^8^, ^9^, ^10^, ^11^

These therapeutic approaches contribute to the enhancement of visual function and help slow the progression of certain ocular diseases. The visually impaired population is predominantly elderly, and neuroretinal pathologies frequently coexist with cataracts at various stages of development.

For example, AMD, the leading cause of low vision among the elderly, is responsible for visual impairment in 10% of individuals aged 65 and older, and in 30% of those over 75 years. Among individuals aged 64 and above with AMD, vision impairment occurs in ~74% of cases.^12^, ^13^ Furthermore, younger patients experiencing visual impairment due to HM or retinitis pigmentosa (RP) may require phacoemulsification surgery to address cataract development.

Phacoemulsification, when combined with the implantation of an appropriately selected intraocular lens (IOL), has the potential to enhance visual acuity in patients with visual impairments.^14^ Therefore, comprehensive guidelines for cataract surgery in patients with low vision would be beneficial to ensure optimal surgical outcomes by improving surgical planning, execution, and postoperative care, along with a well‐coordinated rehabilitation process.^15^, ^16^

The retina is a complex microenvironment in which light is converted into neuroelectric stimuli, subsequently interpreted by the occipital cortex.^17^, ^18^ To effectively process action potentials, the retina requires functional cells characterized by:

- Adequate blood flow.
- An optimal microenvironment devoid of inflammatory cytokines.
- A balanced supply of glucose and oxygen.
- Proper hydration of both the intra‐ and extracellular matrices.
- Sufficient mitochondrial presence.
- Effective signaling pathways.
- Accurate transcription of DNA and subsequent protein synthesis at the ribosomal level.

The retina is characterized by a highly oxidative environment due to its exposure to light and its dependence on oxidative phosphorylation for energy production. This oxidative metabolism results in the formation of reactive oxygen species (ROS), which can interfere with visual function, cellular metabolism, gene transcription, immune responses, and vascular regulation within certain physiological limits.^19^ While ROS play significant roles in normal physiological processes, excessive production can lead to cellular damage and contribute to the onset or progression of various retinal pathologies.^20^

This can lead to the accumulation of polyunsaturated fatty acids (PUFAs), abnormal autophagic processes, subendothelial amyloid deposits, complement dysregulation, and altered gene transcription.^21^, ^22^, ^23^, ^24^ The resulting damage to cytoplasmic membranes and DNA triggers parainflammation and apoptosis, with inflammatory cells, including microglia, being chemotactically recruited. This state can become chronic, characterized by further metabolic decline, increased oxidative imbalance, and progressive pathology.^25^, ^26^

Stabilizing the retina is crucial, particularly in pathologies that can be complicated by exudation, such as DR and AMD. Before surgical intervention, it is crucial to evaluate the most appropriate intravitreal therapies, which may comprise anti‐vascular endothelial growth factor (VEGF) agents, corticosteroids, and topical non‐steroidal anti‐inflammatories (NSAIDs) to effectively manage inflammation. Individuals suffering from visual impairments rely heavily on cells and areas with the highest residual functionality. Cataract surgery can improve the focus on these areas, improving the quality of the reading field and reducing the perception of scotoma size.

Visual rehabilitation employs specialized tools, including microperimetry, prisms, lenses, electronic devices, and targeted neuromodulation techniques that stimulate both the brain and neuroretina. This study aims to establish potential guidelines for achieving optimal rehabilitation outcomes based on a thorough analysis of the existing literature.

## 2 METHODS

A comprehensive literature review of peer‐reviewed articles was conducted using the PubMed database between January 2000 and January 2023, emphasizing recent research on low vision and cataract surgery, visual rehabilitation, and medical retina. Keywords included “Low vision”, “cataract surgery,” “neuroretinal diseases,” “retinal degeneration,” “phacoemulsification,” “visual rehabilitation.” Additional studies were retrieved through citation mapping and hand‐searching of study references.

## 3 RETINA STABILIZATION

Given the role of redox balances in both healthy and pathological retinas, prioritizing neuroretinal stabilization before cataract surgery is essential.

First, breaking the cycle of oxidative imbalance through the administration of essential nutrients, including vitamins A, E, and C, as well as minerals, such as copper, selenium, and zinc is important. Additionally, the use of NSAIDs or other agents may help mitigate retinal parainflammation prior to surgery.^27^, ^28^, ^29^, ^30^ Furthermore, it is important to evaluate the most suitable intravitreal therapies, including anti‐VEGF agents or corticosteroids, along with topical NSAIDs, to effectively manage inflammation.^31^, ^32^, ^33^, ^34^ In specific cases, autologous mesenchymal implants have shown moderate efficacy in treating initial atrophic forms of neuroretinal degeneration.^10^, ^11^, ^35^, ^36^, ^37^ The use of microperimetry to study fixation sensitivity and stability can be more predictive than perimetric analysis for evaluating the impact of phacoemulsification on postoperative visual impairment. In addition, retinography and optical coherence tomography (OCT) are fundamental diagnostic tools for evaluating maculopathy, retinal complications, and optic pathologies. General guidelines for performing cataract surgery on visually impaired patients are given in Box 1. The indications must be adapted and modified individually for each patient to be operated on.

### 3.1 Cataract and age‐related macular degeneration (AMD)

**FIGURE 1:**
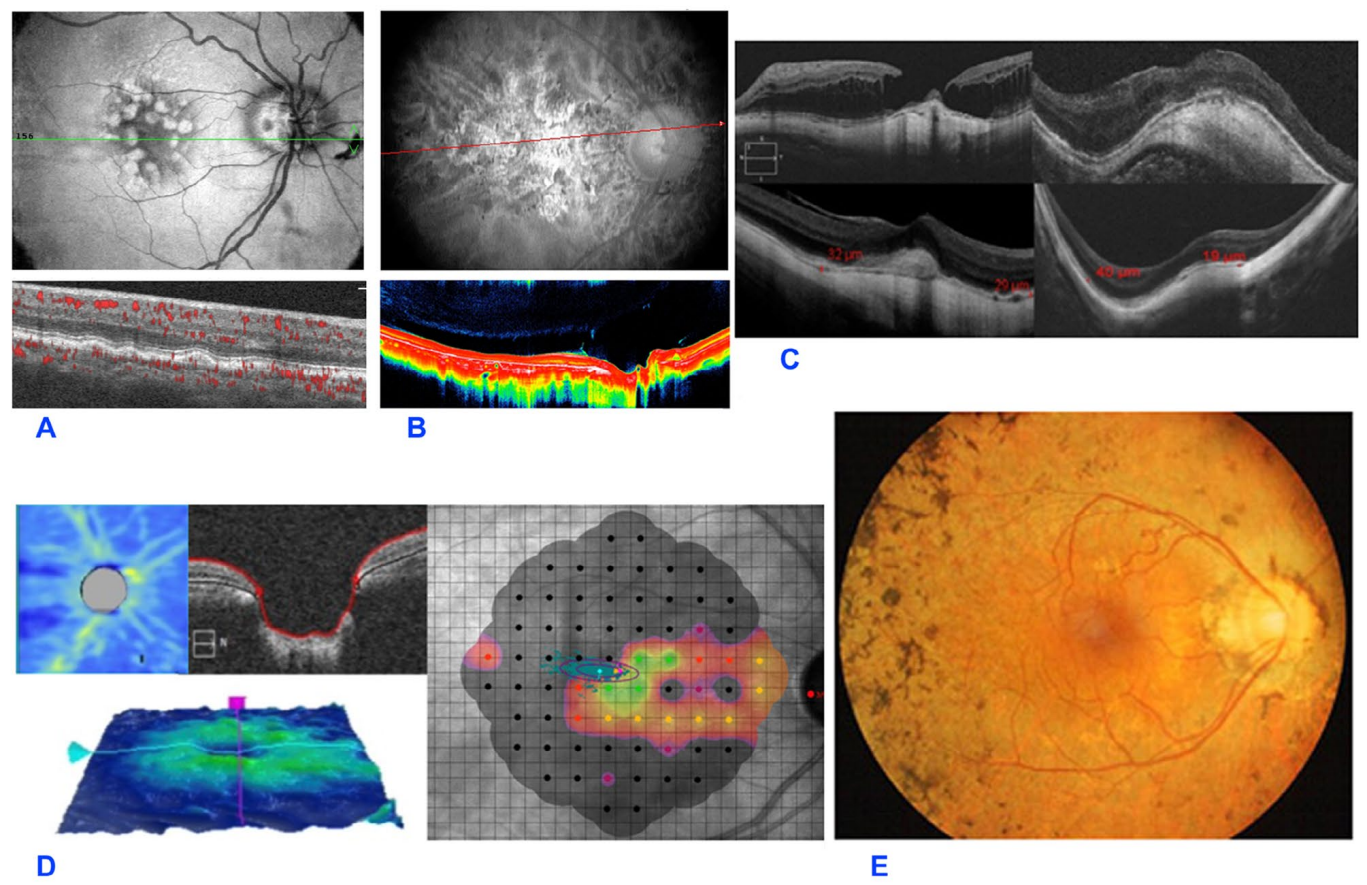
Age‐related macular degeneration (AMD) of colloid type with deposits of lipofuscin material on the outer retina (A). Dry AMD with extensive chorioretinal atrophy (B). Optical coherence tomography (OCT) of patients with various forms of high myopia (HM) chorioretinitis (C). Optic nerve with increased excavation and reduction of sensitivity to microperimetry in glaucoma (GL) patient (D). Retinography of a patient suffering from retinitis pigmentosa with pigment arranged to a spicule‐like pattern (E).

Regarding the potential for exacerbating retinal pathology following cataract surgery, it is important to note that epidemiological studies conducted prior to the introduction of anti‐VEGF therapy demonstrated an increased risk of both exudative and atrophic forms of AMD, including heightened incidence and progression post‐surgery (Figure 1A,B).^38^, ^39^, ^40^, ^41^

Currently, there is no conclusive evidence indicating that cataract surgery increases the incidence or progression of AMD, nor does it facilitate the transition from atrophic to neovascular forms of the disease.^42^, ^43^, ^44^ The retinal condition should be carefully assessed before surgery, ensuring that the macula is stable, whether in an atrophic or exudative state, before scheduling the procedure. In all instances, it is advisable to minimize oxidative stress and retinal inflammation levels. For exudative maculopathies, scheduling the appropriate anti‐VEGF or steroid therapy to reduce macular edema is essential to ensure surgery takes place during reduced disease activity.^45^

Cataract surgery should be performed at least 6 months after starting anti‐VEGF therapy to reduce the risk of short‐term recurrences. Patients with stable eyes and dry retinas in the 6 months before surgery experienced fewer post‐surgery reinjections and a longer time until their first retreatment.^46^, ^47^ However, over a 1‐year follow‐up, these patients did not require more injections than those with AMD who did not have surgery.^48^ Consequently, patients should be properly informed about the need for ongoing monitoring.

### 3.2 Cataract and high myopia (HM)

High axial myopia is defined as a refractive error exceeding −8.00 diopters or an axial length greater than 26.5 mm.^49^ It is associated with anatomical changes in the posterior pole of the eye, including posterior staphylomas and marked choroidal insufficiency, underpinning atrophic and neovascular issues (Figure 1C). Other major pathological changes include scleral thinning, weak zonules of the lens, vitreous opacities, foveal schisis, macular holes, and retinal and choroidal atrophy.^50^

High axial length can impact cataract surgeries through multiple mechanisms. During the procedure, surgeons may face challenges such as lax zonules and fluctuations in anterior chamber depth. These variations can lead to loss of focus, thereby increasing the risk of intraoperative posterior capsule rupture and subsequent retinal breaks. Postoperatively, patients may develop capsular contraction syndrome, which can result in unpredictable refractive outcomes.^51^, ^52^, ^53^

Before proceeding with surgical intervention, it is crucial to stabilize the retina by enhancing choroidal blood flow through the use of prostaglandins, hemorheological agents, electromagnetic stimulation, and growth factors. A thorough assessment of the retinal periphery is essential before surgery, immediately after the procedure, and during subsequent follow‐up visits. In cases of rhegmatogenous lesions, implementing appropriate prophylactic retinal laser barrage is critical.

During surgery, the careful removal of viscoelastic material is critical to prevent pressure spikes and subsequent reductions in choroidal blood flow, which is already compromised in HM.

The ocular kinematics influenced by the elongation of extraocular muscles in highly myopic eyes can lead to postoperative diplopia, particularly due to retinal inhomogeneity and the potential absence of corresponding points. An orthoptic evaluation before and after surgery is beneficial, especially when considering the induction of slight surgical myopia.

In summary, when appropriate precautions are taken, cataract surgery in highly myopic individuals can significantly enhance visual performance, and reduce reliance on thick, heavy corrective lenses.

### 3.3 Cataract and diabetes mellitus

In diabetic patients, hyperglycemia can lead to increased levels of advanced glycosylation end products (AGEs), protein kinase C (PKC), insulin‐like growth factor (IGF), and VEGF, which collectively elevate oxidative stress and contribute to disease progression.^54^, ^55^ Surgical procedures on the eye can further increase stress on the iris, ciliary bodies, and vitreous, resulting in the release of inflammatory cytokines such as interleukin‐1 (IL‐1), interleukin‐6 (IL‐6), tumor necrosis factor‐alpha (TNF‐α), and VEGF. This inflammatory response can exacerbate vascular leakage in the blood‐retinal barrier, which is already compromised due to high oxidative stress.

Studies indicate that ~70% of cases of diabetic macular edema (DME) worsen, with the most significant deterioration occurring within the first month postoperatively.^56^, ^57^ Negative prognostic OCT and OCT‐A biomarkers for these patients include reduced perfusion of the internal and external macular capillary plexus, the presence of hyper‐reflective retinal spots (HRS), serous retinal detachment, irregularities in the ellipsoid zone (EZ), and a central retinal thickness ≥600 microns. To mitigate intraretinal fluid leakage, several treatment options can be utilized, including NSAIDs, anti‐VEGF agents targeting vascular permeability, slow‐release intravitreal steroids, and intraoperative procedures.^58^, ^59^, ^60^, ^61^

### 3.4 Cataract and glaucoma (GL)

GL surgery may lead to cataract formation (Figure 1D).^62^ Recent findings regarding cataract surgery in patients with open‐angle GL, ocular hypertension, and closed‐angle GL have shown a sustained reduction in intraocular pressure (IOP) ranging from 2 to 4 mmHg, lasting for at least 3 years post‐cataract surgery.^63^, ^64^, ^65^

Performing cataract surgery prior to GL surgery presents multiple advantages; specifically, cataract surgery following trabeculectomy increases the risk of subsequent filtration failure unless antimetabolites such as mitomycin are employed.^66^, ^67^, ^68^ Additionally, phacoemulsification increases anterior chamber depth, which aids in the placement of subsequent valve implants IOP management.^68^, ^69^

Given the presence of glaucomatous optic neuropathy, it is advisable to enhance ocular blood flow before, during, and after surgical procedures. This can be achieved through the use of hemorheological agents, prostaglandins, electromagnetic stimulation, and mesenchymal secretome. Moreover, meticulous removal of viscoelastic material during surgery is critical to prevent IOP spikes that could further compromise the already vulnerable optic nerve.

### 3.5 Cataract and inherited degenerative ocular diseases

Cataract, predominantly posterior subcapsular, is a common complication of inherited degenerative ocular diseases, occurring in the mid‐stage of the disease course with evident clinical symptoms and signs (Figure 1E). The main symptom is glare, especially in bright lighting conditions.^70^ Notably, even a small area of lens opacity can lead to a disproportionately significant decline in visual acuity, exacerbated by pre‐existing constriction of the visual field in individuals with inherited degenerative ocular conditions.^71^ Surgery for RP is done earlier, typically between 30 and 63 years of age, compared with age‐related cataract surgery, which is done at an average age of 72–74 years.^70^, ^72^, ^73^, ^74^

RP involves a para‐inflammatory is associated with a para‐inflammatory response that may lead to various complications, including cystoid macular edema, alterations in the vitreoretinal interface, and the development of lamellar holes. RP patients are at a higher risk of intra‐ and postoperative complications, such as phototoxic retinal damage during surgery, early posterior capsular opacification (PCO), capsular contraction syndrome (CCS), pseudophakic cystoid macular edema (PCME), increased postoperative IOP, and IOL dislocation.^75^

Monitoring for surgical complications in patients with RP is crucial.^10^, ^76^, ^77^, ^78^ Preoperative visual acuity and a preserved ellipsoidal area are associated with favorable postoperative outcomes, whereas macular thinning is linked to poorer prognoses.^74^, ^75^, ^76^, ^77^, ^78^, ^79^ The effect of light exposure on the retina following cataract surgery remains unclear;^79^ some studies indicate no correlation between phacoemulsification and increased progression of RP, which appears to depend primarily on the genetic variant.^80^, ^81^

## 4 INTRAOCULAR LENS (IOL) SELECTION

Phacoemulsification is a surgical procedure that involves the removal of the natural lens and the implantation of an artificial IOL to restore visual acuity. In the context of visual rehabilitation, magnifying optical aids are crucial for optimizing visual function and compensating for residual impairments. IOLs are typically utilized to provide optimal natural vision at a distance; however, recent trends have shifted towards premium lenses that enhance vision at intermediate and near distances. In the case of visually impaired patients, a monofocal IOL is usually used.^6^, ^82^, ^83^

IOLs can contribute to facilitating near vision for visually impaired patients by modulating the power of the magnifying system in front of the eye to enable greater image magnification.^84^, ^85^, ^86^

The key parameters for determining the optical aid necessary to facilitate the reading process in individuals with visual impairment include:

- Required magnification.
- Desired field of view: it is inversely related to magnification.
- Desired working distance though it also inversely correlates with magnification.
- Optimal hypercorrective power expressed in diopters.
- IOL selection criteria should consider factors such as asphericity, toricity, and filtration.

### 4.1 Asphericity

The implantation of aspheric IOLs is designed to neutralize the positive corneal aberrations. The reduction of aspheric interferences can improve reading performance. This becomes particularly important in the macular diseases: the asphericity of the IOL makes visual perception easier with areas eccentric to the macular lesion.

### 4.2 Toricity

Astigmatism correction is typically considered when it equals or exceeds one diopter.

The use of toric IOLs to correct astigmatism enables the elimination or reduction of astigmatic aberrations. This correction is particularly beneficial for patients with macular degeneration who depend on eccentric vision, as it maintains visual clarity even during eye movements.

### 4.3 Light filtration

The natural crystalline lens serves as a protective barrier, filtering harmful ultraviolet (UV) and blue light energy. The surgical removal of the crystalline lens increases the transmission of these light wavelengths, particularly in the spectral range of 300–700 nm, which includes visible blue light. Acute exposure to UV radiation and high‐energy blue light can lead to photochemical damage to the retina. To mitigate this risk, Blue Light Filtering IOLs, commonly referred to as “yellow lenses,” are designed with chromophores that absorb high‐energy blue radiation. Their transmittance curve approximates that of a typical lens from a 50‐year‐old individual.^6^, ^82^, ^83^, ^86^

### 4.4 Choosing the correction for post‐surgery: emmetropization versus myopization

Patients who opt against wearing distance lenses post‐cataract surgery will continue using their pre‐existing visual aids, such as electronic video magnifiers, to facilitate reading. Notably, individuals with myopia and macular damage often benefit from a myopic correction, as it allows them to bring text closer, thus exploiting the natural magnification effect associated with myopia. For instance, a patient with a −10 diopter (D) prescription can read at a distance of 10 cm, achieving a magnification factor of ~2.5 times. This adjustment can help compensate for scotomas resulting from chorioretinal atrophy in the macula.

An optimal myopic correction of around −2.5 to −3 D is recommended, as it minimizes the likelihood of postoperative overcorrection while providing sufficient visual autonomy in daily activities, requiring glasses mainly for outdoor use. Additionally, distance glasses may be enhanced with photo‐selective filters to improve contrast and reduce oxidative stress caused by light exposure.

### 4.5 Choosing the type of IOL: monofocal, multifocal, or continuous focus IOL

*Monofocal IOLs* are primarily used when the main objective is to achieve emmetropization for optimal distance vision. In patients with low vision, monofocal IOLs can be employed to induce surgical myopization, enhancing near or intermediate vision capabilities. For individuals with severe visual impairments, emmetropization is often favored, as reading is typically facilitated only through the use of electronic video magnifiers. Monofocal IOLs are also indicated in cases where significant corneal aberrations exist, such as those resulting from previous refractive surgeries, corneal transplants, keratoconus, or substantial astigmatism. In these scenarios, toric monofocal IOLs are utilized to correct astigmatism while providing improved visual outcomes.

*Multifocal IOLs* facilitate vision at various distances, enhancing the ability to focus on both near and far objects. However, there is limited research on the efficacy of multifocal lenses in patients with maculopathy, as previous studies have often excluded eyes with neuroretinal pathologies that could confound the evaluation of visual parameters.^5^, ^12^, ^87^, ^88^

This feature allows for functional near vision even in suboptimal lighting situations. Currently, hydrophobic acrylic materials with yellow chromophores are the most commonly utilized for these lenses, as they mimic the human crystalline lens and effectively filter harmful wavelengths that could reach the retina.^89^

The reduced dependence on corrective eyewear afforded by multifocal IOLs enables visually impaired individuals to utilize less powerful visual aids, ultimately enhancing their compliance and satisfaction with reading activities.^90^

*Continuous focus IOLs*, also known as Extended Depth of Focus (EDoF) lenses, represent a feasible alternative to traditional multifocal lenses. EDoF technology employs controlled spherical aberration to create a continuous focus, effectively increasing the depth of field through advanced wavefront technology.^91^ This innovation enables clear vision with minimal visual disturbances, allowing for a halo‐free experience at distances ranging from approximately 55 cm to infinity.^92^, ^93^, ^94^

In patients with visual impairments, EDoF lenses can serve as an excellent compromise, particularly when significant hypercorrection is unnecessary.

*Telescopic IOLs* have been developed to aid patients with AMD, including the implantable miniaturized telescope (IMT), the IOL‐VIP system, the EyeMax Mono system, and the Scharioth Macular Lens (SML). Among these, the IMT is the only device approved by the FDA in the United States, designed to enhance central vision while compromising peripheral vision. The implantation of the IMT requires a larger incision, necessitating 6–8 sutures for closure, which may lead to significant postoperative astigmatism. The IOL‐VIP and EyeMax Mono systems feature smaller incisions and are applicable to both phakic and pseudophakic eyes, offering similar levels of magnification. A notable advancement with the EyeMax Mono system is its ability to deliver high‐quality images across all macular regions up to 10° from the fovea, making it advantageous in cases where there are changes in the preferred retinal locus (PRL) or progression of AMD.

Although the SML does not provide magnification for distance vision, it can magnify objects by a factor of two within a 10–15 cm range from the eye and can be implanted through a smaller 2.2 mm incision, thereby reducing the risk of postoperative astigmatism.

However, it is critical to consider ocular anatomy when selecting a telescopic IOL, as these devices may lead to complications such as pupillary block and increased IOP. Additionally, the alteration in environmental proportions necessary to achieve sufficient magnification for reading may impact daily life.^94^, ^95^, ^96^, ^97^

## 5 CONSIDERATIONS DURING SURGERY

The microscope light used during cataract surgery can increase retinal oxidation, especially after cataract removal. To minimize oxidative damage, it is crucial to reduce surgical times. High‐tech phacoemulsification devices utilize less ultrasonic energy, and the “Ozil” torsional phaco tip, which vibrates by rotating, significantly enhances the technique's efficiency. In selected cases, such as intermediate cataracts, femtosecond laser‐assisted cataract surgery can yield results comparable to traditional ultrasound techniques. Modern phacoemulsifiers come equipped with sensors that control irrigation, aiding in maintaining IOP. The small 2.2 mm incision of the main entry site facilitates faster recovery and better pressure control, which is particularly important in cases with exudative backgrounds, as postoperative IOP should not be excessively low. Proper removal of the viscoelastic material used during surgery is crucial to preventing hypertensive spikes that could diminish retinal perfusion.

Inflammation can be managed by employing NSAIDs both preoperatively and postoperatively. However, the release of chemotactic and vasoactive cytokines may promote the recurrence of neovascular pathology or lead to cystoid macular edema. In atrophic forms, surgeries may be preceded by treatments that enhance neuroretinal health, such as neuroprotective agents. For higher risk cases, a bolus of intravenous or subconjunctival steroids may be beneficial immediately after surgery. Additionally, intravitreal treatment with anti‐VEGF agents or slow‐release dexamethasone can be administered right after the operation to help reduce inflammation and complications.

In summary, careful surgical techniques, preoperative stabilization, and effective inflammation management are critical to ensure positive outcomes and mitigate the impact of surgery on potential neuroretinal pathologies.

## 6 CONSIDERATIONS AFTER SURGERY

The postoperative period is critical and requires careful monitoring, especially for patients with visual impairments. To ensure timely intervention, patients should have a contact number to report sudden changes and arrange follow‐up appointments for monitoring treatable alterations.

Surgical interventions can activate inflammatory and vasoactive cytokines, destabilizing the retina in visually impaired individuals. An effective therapeutic approach involves a combination of anti‐inflammatory treatments, such as NSAID eye drops, administered for a minimum of 1–2 months, along with antibiotic‐steroid therapy limited to no more than 10 days. NSAID eye drops are preferred due to their superior retinal penetration compared with steroids and their minimal impact on IOP. Sudden increases in IOP may occur as a result of steroid therapies or the presence of residual viscoelastic material in the anterior chamber.^98^, ^99^ Diabetic patients should adhere to recommended blood glucose levels, while hypertensive individuals need to monitor their blood pressure.^100^, ^101^, ^102^

Ten days post‐surgery, patients can resume any supplements they were using prior to the operation, as these can help maintain stable retinal conditions due to their antioxidant, anti‐inflammatory, and trophic properties.^103^, ^104^ For patients with a history of exudative conditions, it is vital to schedule anti‐VEGF therapy immediately upon recurrence. This underscores the importance of postoperative monitoring through imaging techniques to detect potential neovascular recurrence, subretinal or intraretinal fluid, and pseudophakic macular edema, which may complicate the clinical picture.

The Amsler grid test is recommended both before and after surgery, enabling patients to report any changes. Microperimetric analysis of sensitivity should be conducted within 1–3 months post‐surgery to evaluate the stability of the underlying neuroretinal pathology and the effectiveness of the intervention. The rehabilitation process is central for visually impaired patients to regain reading capabilities as soon as possible, with our experience suggesting that this can commence as early as 3 weeks after the surgical procedure. Even if best‐corrected visual acuity (BCVA) remains unchanged, the functional condition of the patient may significantly improve compared with the preoperative state.

Overall, meticulous management of the postoperative period is essential to minimize complications and enhance visual and functional outcomes for visually impaired patients.

## 7 CONCLUSION

In conclusion, the evaluation of patients with cataracts and low vision necessitates careful consideration of several critical factors. Cataract surgery generally leads to significant improvements in patients' quality of life, and there should be no undue hesitation in recommending the procedure. Ultimately, the management of patients with cataracts and maculopathies extends well beyond the surgical intervention itself. It encompasses comprehensive preoperative assessments, meticulous postoperative follow‐up, and individualized rehabilitation strategies aimed at addressing each patient's unique needs. By emphasizing informed consent and careful monitoring, healthcare providers can improve visual outcomes and significantly enhance patients' quality of life.

## AUTHOR CONTRIBUTIONS

Conceptualization, methodology, and writing original draft preparation, P.G.L.; validation, formal analysis, investigation, C.L.; resources, data curation, writing review and editing, M.N. All authors have read and agreed to the published version of the manuscript.

## FUNDING INFORMATION

This research received no external funding.

## CONFLICT OF INTEREST STATEMENT

The authors declare no conflicts of interest related to this study.

## ETHICS STATEMENT

This study is based on data obtained from the International scientific literature.

BOX 1 **A synopsis of the working procedure for cataract surgery and rehabilitation in visually impaired patients**

**Patient Selection and retina stabilization**:

- Visually impaired patients require careful monitoring of low vision conditions and constant follow‐up of their retina.
- Cataract surgery should be considered if there is a progressing cataract and stable retina.

**Surgery:**

- The procedure should minimize retinal light exposure.
- It is essential to know the patient's rehabilitation possibilities
- The intraocular lens (IOL) should be selected based on visual rehabilitation needs, including a yellow filter.
- Preventative measures against inflammation are essential.

**Postoperative Care:**

- Continuous monitoring through OCT, microperimetry, and tonometry is critical.
- Anti‐inflammatory therapy should last beyond the first month, with possible use of carbonic anhydrase inhibitors.

**Rehabilitation Phase:**

- After 20–30 days, reassess visual aids considering IOL characteristics (surgical myopization, emmetropization, depth of field).
- Neuromodulation cycles may be necessary to enhance neuroretinal function and stabilize fixation on the Preferential Retinal Locus (PRL).

*Risk Mitigation Strategies before and after surgery to manage oxidative stress:

Enhancing antioxidant capacities with the administration of lutein, betulinic acid, glutathione, neurotrophic substances, growth factors, cell therapies, and similar substances.

*Multidisciplinary Approach for a model for holistic patient care:

It is recommended the involvement of a multidisciplinary team in the surgical and rehabilitation process, encompassing ophthalmologists, neurologists, optometrists, and rehabilitation specialists.
